# PrEP preferences and early acceptability of injectable cabotegravir among pregnant and lactating people in Cape Town, South Africa: findings from the PrEPared to Choose study

**DOI:** 10.1002/jia2.26492

**Published:** 2025-07-02

**Authors:** Nafisa Wara, Carey Pike, Elzette Rousseau, Pippa Macdonald, Pakama Mapukata, Bryan Leonard, Keitumetse Lebelo, Risa Hoffman, Catherine Orrell, Linda‐Gail Bekker, Dvora Joseph Davey

**Affiliations:** ^1^ Los Angeles David Geffen School of Medicine University of California Los Angeles California USA; ^2^ Desmond Tutu HIV Centre University of Cape Town Cape Town South Africa; ^3^ Division of Infectious Diseases Los Angeles David Geffen School of Medicine University of California Los Angeles California USA; ^4^ Division of Epidemiology and Biostatistics University of Cape Town School of Public Health Cape Town South Africa

**Keywords:** CAB‐LA, lactation, pregnancy; breastfeeding, PrEP, South Africa

## Abstract

**Introduction:**

Providing pregnant and lactating people (PLP) with choice in HIV pre‐exposure prophylaxis (PrEP) methods, including long‐acting injectable cabotegravir (CAB‐LA), may mitigate barriers to effective PrEP use. We evaluated PrEP preferences and acceptability among PLP offered CAB‐LA versus oral PrEP in South Africa.

**Methods:**

The PrEPared to Choose study in Cape Town, South Africa, enrolled young people ages 15–29 at one public clinic and one community‐based mobile clinic. Using informed choice counselling, participants were offered oral PrEP or CAB‐LA, with the option to switch methods at follow‐up visits over 18 months. We report baseline CAB‐LA and oral PrEP initiations among PLP in the study, acceptability of their initial choice within 3 months of enrolment and theoretical preferences regarding PrEP methods that may become available to PLP. We report descriptive statistics and use Chi‐square and Fisher's exact to compare responses by initiated PrEP method and pregnancy status.

**Results:**

From February to August 2024, we enrolled 58 PLP (*n* = 30 pregnant, *n* = 28 breastfeeding). Median age 23 years (IQR 19.5−26). Of 30 pregnant participants, 23 (77%) initiated CAB‐LA and seven (23%) oral PrEP; among 28 breastfeeding participants, 25 (89%) initiated CAB‐LA and three (11%) oral PrEP. Of enrolled PLP, 36 (62%, *n* = 13 pregnant, *n* = 23 breastfeeding) completed the acceptability survey. Of these, 83% (*n* = 12/13 pregnant, *n* = 20/23 breastfeeding) chose and received CAB‐LA, and the remaining (*n* = 4) chose and received oral PrEP. PLP who received CAB‐LA reported liking its ease of use (69%; *n* = 22/32) and long‐acting protection (44%; *n* = 14/32). Half of CAB‐LA users disliked side effects (e.g. injection site pain), although 41% of PLP (*n* = 13/32) described no CAB‐LA dislikes. Almost all (97%; *n* = 31/32) PLP currently using CAB‐LA were interested in continuing CAB‐LA, and all PLP using oral PrEP reported interest in trying CAB‐LA in the future. Eighty‐six percent of surveyed PLP (*n* = 31/36) did not want to try the dapivirine vaginal ring.

**Conclusions:**

PLP in South Africa had a strong preference for CAB‐LA over oral PrEP, and CAB‐LA was found to be highly acceptable. Further research is needed to evaluate the effect of offering choice of PrEP methods, including CAB‐LA, on PrEP continuation among PLP.

## 1 INTRODUCTION

Adolescent girls and young women who are pregnant or breastfeeding are at increased risk of HIV acquisition [1, 2]. HIV acquisition risk increases by > 2‐fold during pregnancy and postpartum [3, 4], and acute maternal HIV acquisition is associated with a significantly increased risk of vertical HIV transmission [5, 6, 7], making primary HIV prevention in pregnant and lactating people (PLP) a global health priority [8, 9]. Oral pre‐exposure prophylaxis (PrEP), an effective daily HIV prevention method, has been scaled in several African countries with high HIV burden, including South Africa, with over 1.8 million oral PrEP initiations through 2024 [10]. Ensuring effective PrEP use among PLP will accelerate progress to achieving UNAIDS and South Africa's goal of eliminating vertical transmission by 2025 [11].

New PrEP methods, including long‐acting injectable cabotegravir (CAB‐LA) and the dapivirine vaginal ring (DVR), are effective methods of HIV prevention, with a safety profile that is consistent with that established in non‐pregnant people, and are becoming available in African countries, including South Africa, Zimbabwe, Zambia, Botswana and Malawi [12, 13, 14, 15, 16, 17]. CAB‐LA, an integrase inhibitor administered via intramuscular injection every 8 weeks to prevent HIV acquisition, has shown high efficacy, safety and superiority over oral PrEP formulations in male and female global cohorts [18, 19]. CAB‐LA reduces HIV acquisition risk by 89% compared to oral PrEP in women in sub‐Saharan Africa [20]. Although recent studies have found DVR to be safe and effective for use in pregnancy, it is not yet approved for use in pregnancy in South Africa [21, 22].

Choice in PrEP methods has been shown to improve PrEP continuation in cisgender women, yet is not part of standard care. Initial results from an open‐label extension of HPTN‐084 demonstrated that in 1000 women, 78% chose to start or continue CAB‐LA, and 68% of 233 pregnant women chose to take CAB‐LA. Product choice was influenced by personal preference for product attributes, social context and risk behaviours; participants expressed limited decisional conflict [23]. In the MTN‐034/REACH trial among non‐pregnant African young women, adherence was high in those given the choice between oral PrEP and DVR, with high adherence validated with objective measures [14]. Results from the SEARCH study in Kenya and Uganda demonstrated that dynamic choice in HIV prevention methods and delivery strategies led to a 56% increase in time covered by a biomedical PrEP product, including CAB‐LA [24]. Despite these reassuring results, PLP have not had a choice of PrEP methods, such as CAB‐LA, outside of clinical trials due to slow progress towards universal access and limited regulatory approval for use by this population.

The critical need to prevent HIV acquisition in PLP and vertical HIV transmission provides a strong rationale for accelerating PLP access to new methods, such as CAB‐LA, alongside counselling and the ability to switch to ensure persistence across pregnancy and postpartum periods [8]. We evaluated early data from PLP enrolled in the PrEPared to Choose study, an ongoing implementation study utilizing informed choice counselling to offer choice in PrEP methods (injectable, vaginal and oral) in Cape Town, South Africa [25, 26]. In this manuscript, we report baseline PrEP method selection from all PLP enrolled in the study, and acceptability data from PLP who returned to complete an additional survey within 3 months of study enrolment.

## 2 METHODS

### 2.1 Study participants

The PrEPared to Choose study is an ongoing implementation study evaluating the delivery of CAB‐LA among adolescents and young people ages 15–29 years living within the Klipfontein‐Mitchell's Plain sub‐districts of Cape Town, South Africa [27]. The study enrolled eligible, consenting participants in one government clinic and one mobile clinic offering PrEP and sexual health services as part of outreach services and ongoing research studies. Eligible participants interested in starting PrEP received informed choice counselling and were offered oral tenofovir disoproxil fumarate/emtricitabine (TDF/FTC) PrEP, injectable CAB‐LA and DVR [25, 26]. Participants were evaluated for clinical eligibility for their chosen PrEP product, and if eligible, initiated on their chosen PrEP method, with an option to switch at all future study visits. Participants returned for study visits as required for clinical follow‐up (e.g. every 2 months if using CAB‐LA, every 3 months if using oral PrEP or the DVR) over 18 months, and follow‐up is ongoing. The PrEPared to Choose study is nested within FastPrEP, an ongoing demonstration project scaling current standard of care PrEP methods through the utilization of novel community‐ and clinic‐based differentiated service delivery strategies [28].

As part of PrEPared to Choose, PLP were included and offered oral PrEP and CAB‐LA per South African clinical guidelines (DVR was not offered to PLP due to contraindication during pregnancy and breastfeeding pending review of updated safety data) [29, 30]. Inclusion criteria for PLP enrolled into PrEPared to Choose were: (1) ages 15–29 years; (2) HIV negative at baseline as per national clinical testing guidelines; (3) ≥ 35 kg body weight at baseline; (4) currently residing in the study area; and (5) able to provide written, informed consent.

### 2.2 Data collection

Enrolment for PrEPared to Choose took place between February and August 2024. Trained study staff fluent in English and isiXhosa introduced the study to pregnant and breastfeeding participants accessing health services at the government clinic or mobile clinic. Study staff screened for study eligibility, obtained written informed consent in English or isiXhosa (including waiver of parental consent for 15‐ to 17‐year‐olds), and completed baseline study activities, including collection of demographic data, provision of informed choice counselling and offer of oral TDF/FTC versus CAB‐LA with relevant clinical laboratory testing per South African clinical guidelines. From May to October 2024, the study staff asked all pregnant and breastfeeding participants at the baseline visit if they were interested in completing an additional acceptability study. For PLP already enrolled in the main study prior to May 2024, the same option was offered via phone call or at the next follow‐up visit. If the participant was interested, they were referred to research assistants fluent in English and isiXhosa based at a nearby midwife obstetric unit to document informed consent and complete the acceptability survey. The trained research assistants collected responses on a tablet using REDCap, a secure web‐based platform [31, 32]. Baseline procedures took 45–60 minutes and the acceptability survey took 30–45 minutes. Participants received 150 South African Rand (∼$8 USD) for each study visit as well as transportation expenses. No reimbursement was given for other study procedures, including PrEP prescription and provision.

### 2.3 Survey measures

#### 2.3.1 Baseline socio‐demographic characteristics

At the baseline PrEPared to Choose study visit, the following participant characteristics were collected: (1) basic demographic data (including age, obstetric history, education level, relationship status, number of living children, employment); (2) information regarding sexual activity (including partners’ HIV status, condom usage in the last month, sexually transmitted infection (STI) history in the past 1 month); (3) perceived likelihood of acquiring HIV in the previous 3 months; (4) prior PrEP use; (5) depression screening adapted from the Patient Health Questionnaire (PHQ‐4); and (6) alcohol use adapted from the Alcohol Use Disorders Identification Test [33, 34, 35].

#### 2.3.2 Acceptability survey

We asked participants about their current HIV prevention methods, duration of time on PrEP, self‐reported weekly adherence for those taking oral PrEP and reasons for stopping PrEP if applicable. We asked participants who reported using CAB‐LA about their likes, dislikes, comfort with receiving injections, prior experience with injections and willingness to continue using CAB‐LA, informed by domains of the Theoretical Framework of Acceptability [36]. We asked all pregnant participants about willingness to try or continue CAB‐LA after giving birth. We asked all participants who took the acceptability survey, regardless of PrEP use, about their experience with vaginal rings, theoretical likes and dislikes regarding the DVR, comfort with the idea of vaginal ring insertion and theoretical willingness to try the DVR in the future. We also asked all participants about their ideal frequency for receiving a PrEP product, method of insertion, person administering the PrEP injection and location to receive a PrEP product, and we asked them to rank the top two most important features of a PrEP product.

### 2.4 Statistical analyses

We used descriptive statistics (median, interquartile range [IQR], frequency) to report participant responses. We used Chi‐square, Fisher's exact and Wilcoxon rank sum to compare demographic characteristics by PrEP method selection, as well as acceptability survey responses by pregnancy status, with a significance threshold of *p* < 0.05. Statistical analyses were conducted with STATA v.18 [37].

### 2.5 Ethics

The PrEPared to Choose study was approved by the Human Research Ethics Committee of the University of Cape Town Faculty of Health Sciences (HREC 567/2023).

## 3 RESULTS

### 3.1 Demographic characteristics and initial PrEP choice

**Figure 1:**
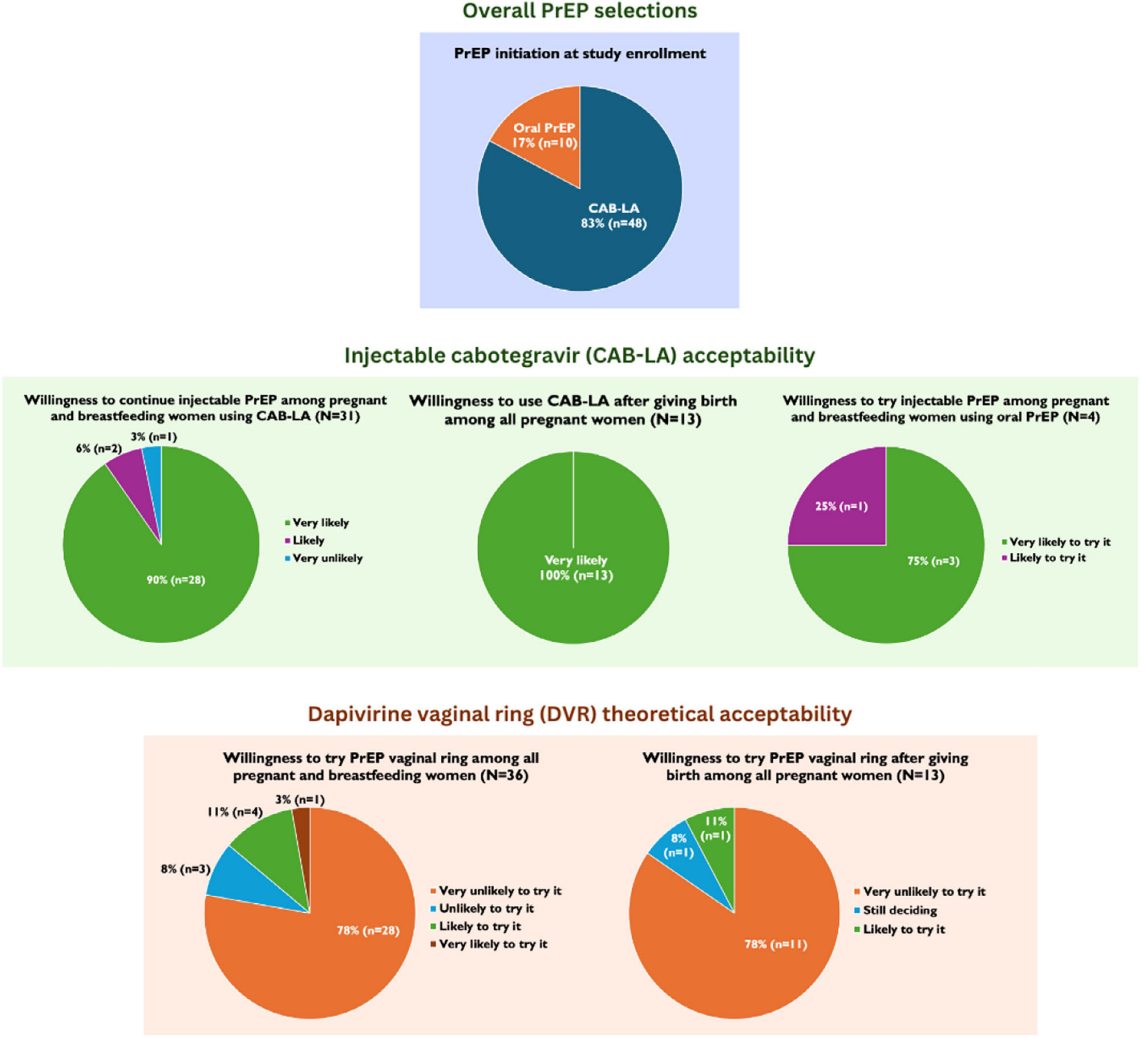
**Pre‐exposure prophylaxis (PrEP) selections and acceptability of long‐acting injectable cabotegravir (CAB‐LA) and dapivirine vaginal ring (DVR) among pregnant and breastfeeding women, Cape Town, South Africa (February 2024–October 2024)**. Of *N* = 58 pregnant and breastfeeding participants, 83% (*n* = 48) chose to initiate CAB‐LA, while the remaining (17%, *n* = 10) started oral PrEP. *N* = 36 pregnant and breastfeeding women chose to return for a follow‐up survey evaluating acceptability of CAB‐LA and the DVR, with key results presented in graphic format. Acceptability questions were asked with Likert scale response options (e.g. “Very likely, likely, unsure, unlikely, very unlikely”).

From February 2024 to August 2024, we enrolled 58 cisgender pregnant or breastfeeding women (26 pregnant, 32 breastfeeding) (Table 1). Median age was 23 years (IQR 19.5−26) and most were enrolled at the government clinic (72%, *n* = 42) versus the mobile clinic. The majority of participants (88%, *n* = 51) reported having a partner or being married, and 64% (*n* = 37) had at least one living child at the time of enrolment. Slightly over half (57%, *n* = 33) were unemployed and not attending school, and 64% (*n* = 37) had completed grade 12 or higher education. Regarding participant sexual history, 52% of participants (*n* = 30) reported that their partner's HIV status was unknown to them, while 45% reported that their partners were living without HIV, and two participants (3%) reported having partners who were living with HIV. Almost all participants (97%, *n* = 56) said that they did not consistently use condoms during sex or were unsure about condom use in the last month, and nearly all participants (93%, *n* = 54) reported that neither they nor their partner had an STI in the last month. When asked about their perceived likelihood of having acquired HIV in the last 3 months, 78% (*n* = 45) described it as a “small chance,” followed by 16% (*n* = 9) who described a “moderate” or “high chance,” and 10% (*n* = 6) who described there being “no chance.” Median PHQ‐4 score was 0 (IQR 0–2). The majority of participants (81%, *n* = 47) reported no previous PrEP use. Of 58 PLP, 48 participants (83%) chose to initiate CAB‐LA, and the remaining 10 (17%) chose oral PrEP (Figure 1). Participants who chose CAB‐LA versus oral PrEP did not significantly differ by any of the aforementioned demographic characteristics.

**Table 1:** Demographic characteristics at enrolment of pregnant and lactating people offered choice in HIV pre‐exposure prophylaxis (PrEP) modalities in Cape Town, South Africa, February 2024–August 2024 (*N* = 58)

PrEP initiation at enrolment	Overall *N* = 58 (%)	CAB‐LA *n* = 48 (%)	Oral TDF/FTC *n* = 10 (%)	*p*‐value
Age (median, IQR)	23 (19.5−26)	23 (19−26)	21 (18.5−27)	0.897
Pregnancy status				
Pregnant	26 (45)	23 (48)	3 (30)	0.487
Breastfeeding/lactating	32 (55)	25 (52)	7 (70)	
Enrolment site				
Clinic	42 (72)	37 (77)	5 (50)	0.12
Mobile van	16 (28)	11 (23)	5 (50)	
Relationship status				
Has partner/married	51 (88)	43 (90)	8 (80)	0.592
Single	7 (12)	5 (10)	2 (20)	
Number of living children				
0	21 (36)	16 (33)	5 (50)	0.471
≥1	37 (64)	32 (67)	5 (50)	
Employment status				
Unemployed	33 (57)	28 (58)	5 (50)	0.732
Employed or attending school	25 (43)	20 (42)	5 (50)	
Highest level of education completed				
Matric (Grade 12) or higher	37 (64)	28 (58)	9 (90)	0.077
Secondary school (Grade 8−11)	21 (36)	20 (42)	1 (10)	
Partner(s)’ HIV status				
Without HIV	26 (45)	21 (44)	5 (50)	0.94
Living with HIV	2 (3)	2 (4)	0 (0)	
Unknown	30 (52)	25 (52)	5 (50)	
Consistent condom use during sex in last 1 month				
No/don't know	56 (97)	46 (96)	10 (100)	1
Yes	2 (3)	2 (4)	0 (0)	
Participant or partner had STI in last 1 month				
No	54 (93)	45 (94)	9 (90)	0.295
Yes	2 (3)	2 (4)	0 (0)	
Don't know	2 (3)	1 (2)	1 (10)	
Perceived likelihood of being infected with HIV in last 3 months^a^				
No chance	6 (10)	5 (10)	1 (10)	0.597
Small chance	45 (78)	34 (71)	9 (90)	
Moderate or high chance	9 (16)	9 (19)	0 (0)	
Prior PrEP use?				
No	47 (81)	39 (81)	8 (80)	1
Yes	11 (19)	9 (19)	2 (20)	
PHQ‐4 (Median, IQR)^b^	0 (0−2)	0 (0−1.5)	1 (0−2)	0.056

Abbreviations: CAB‐LA, long‐acting injectable cabotegravir; IQR, interquartile range; PHQ‐4, Patient Health Questionnaire; PrEP, pre‐exposure prophylaxis; STI, sexually transmitted infection; TDF/FTC, tenofovir disoproxil fumarate/emtricitabine.

^a^ Participants were asked “Right now, how likely do you think it is that you could be infected with HIV (if you didn't use PrEP)?” with the option to select “Very unlikely,” “Unlikely,” “I'm not sure,” “Likely” or “Very likely.”

^b^ Patient Health Questionnaire‐4.

### 3.2 PrEP use among PLP at time of conducting acceptability survey

From May to October 2024, we completed surveys with 38 participants who were enrolled during pregnancy or while breastfeeding into the PrEPared to Choose study within the previous 3 months (Figure S1). Of these, 13 participants were pregnant, 23 were postpartum, one reported termination of pregnancy and one reported no longer being pregnant or postpartum without specifying a pregnancy outcome. At the time of completing the survey, all pregnant participants (*n* = 13) and 96% (*n* = 22/23) of postpartum participants were using some form of PrEP, 64% (*n* = 24) participants reported using condoms and one pregnant participant reported post‐exposure prophylaxis use (Table 2). Of 13 pregnant women, 12 (92%) were using CAB‐LA and one was using oral PrEP, while among 23 postpartum women, 19 (83%) were using CAB‐LA, three (13%) were using oral PrEP and one (4%) initiated CAB‐LA at study enrolment but missed their latest injection appointment due to giving birth. When asked how long the participant had used PrEP (including PrEP use prior to study enrolment), 62% of pregnant women (*n* = 8) and 52% of postpartum women (*n* = 12) reported 1–3 months of PrEP use, followed by < 1 month and > 3 months. Among the four participants who reported using oral PrEP, two participants (one pregnant, one postpartum) said that they took oral PrEP every day, one postpartum woman reported taking 4–6 pills per week and one postpartum participant reported taking 0–1 pill per week.

**Table 2:** HIV pre‐exposure prophylaxis (PrEP) use among pregnant and postpartum women offered long‐acting injectable cabotegravir (CAB‐LA) and oral PrEP in Cape Town, South Africa, May 2024–October 2024

	Total (*N* = 36, %)	Pregnant (*n* = 13, %)	Postpartum (*n* = 23, %)	*p*‐value
Initiated PrEP use at time of completing acceptability survey				
CAB‐LA	31 (86)	12 (92)	19 (83)	0.946
Oral PrEP	4 (11)	1 (8)	3 (13)	
Not using PrEP, but previously used CAB‐LA	1 (3)	0 (0)	1 (4)	
Current HIV prevention methods used at time of acceptability survey				
PrEP (any product)	35 (97)	13 (100)	22 (96)	1
Condoms	24 (64)	10 (77)	14 (61)	0.469
PEP	1 (3)	1 (8)	0 (0)	1
Duration of time on PrEP (including previous PrEP use)				
<1 month	8 (22)	4 (31)	4 (17)	0.255
1−3 months	20 (56)	8 (62)	12 (52)	
>3 months	8 (22)	1 (8)	7 (30)	
If using oral PrEP: self‐reported weekly adherence^a^				
Every day	2/4 (50)	1/1 (100)	1/3 (33)	0.870
4−6 pills per week	1/4 (25)	0 (0)	1/3 (33)	
0−1 pill per week	1/4 (25)	0 (0)	1/3 (33)	
If stopped PrEP: reasons for stopping PrEP use				
Gave birth	1 (100)	0 (0)	1 (100)	1

Abbreviations: CAB‐LA, long‐acting injectable cabotegravir; PrEP, pre‐exposure prophylaxis.

^a^ 2−3 pills per week omitted due to zero responses.

### 3.3 CAB‐LA acceptability

Among 32 PLP who had initiated CAB‐LA at study enrolment, when asked what they liked about CAB‐LA, the most frequent response was its ease of use (69% of participants, *n* = 22), followed by its long‐acting duration of effectiveness (44%, *n* = 14) and being protected from HIV (19%, *n* = 6) (Table 3). While 58% of pregnant women (*n* = 7/12) and 30% of postpartum women (*n* = 6/20) reported no dislikes regarding CAB‐LA, 25% of those who were pregnant (*n* = 3/12) and 65% of those who were postpartum (*n* = 13/20) disliked side effects related to CAB‐LA, such as injection pain. Despite this, 100% (*n* = 12) of pregnant individuals and 95% (*n* = 18/19) of postpartum women using CAB‐LA reported that they were “very likely” or “likely” to keep using CAB‐LA, while one postpartum individual responded that they were “very unlikely” to continue using CAB‐LA. Of the four PLP using oral PrEP, all (100%) reported that they were “very likely” or “likely” to try CAB‐LA in the future. Among surveyed PLP, regardless of their chosen PrEP method, 89% said they were “very comfortable” or “comfortable” with injections, although 53% (*n* = 19) reported never previously using an injection (e.g. contraceptives, vitamin injections, chronic injectable medicines, vaccines). Of 13 pregnant individuals (12 using CAB‐LA and one using oral PrEP), 100% said they were “very likely” to continue or start CAB‐LA after giving birth.

**Table 3:** Acceptability of long‐acting injectable cabotegravir (CAB‐LA) and dapivirine vaginal ring (DVR) among pregnant and postpartum women offered oral pre‐exposure prophylaxis (PrEP) and CAB‐LA in Cape Town, South Africa, May 2024–October 2024

CAB‐LA acceptability among CAB‐LA initiators	Total (*N* = 32, %)	Pregnant (*n* = 12, %)	Postpartum (*n* = 20, %)	*p*‐value
Injectable PrEP likes				
Ease of use	22 (69)	9 (75)	13 (65)	0.703
Long‐acting	14 (44)	5 (42)	9 (45)	1
Protection from HIV	6 (19)	4 (33)	2 (10)	0.165
Discreet (no pill bottle)	4 (13)	2 (17)	2 (10)	0.620
Modality (that it is an injection)	1 (3)	0 (0)	1 (5)	1
Other	5 (16)	1 (8)	4 (20)	0.626
Injectable PrEP dislikes				
Side effects (e.g. injection site reaction)	16 (50)	3 (25)	13 (65)	0.066
Modality (that it is an injection)	2 (6)	2 (17)	0 (0)	0.277
Other	2 (6)	1 (8)	1 (5)	1
Nothing	13 (41)	7 (58)	6 (30)	0.262
Willingness to continue injectable PrEP^a^				
Very unlikely	1/31 (3)	0 (0)	1/19 (5)	0.329
Likely	2/31 (6)	2/12 (17)	0 (0)	
Very likely	28/31 (90)	10/12 (83)	18/19 (95)	

Theoretical CAB‐LA acceptability among oral PrEP users	Total (*N* = 4, %)	Pregnant (*n* = 1, %)	Postpartum (*n* = 3, %)	*p*‐value
Willingness to try injectable PrEP (if using oral PrEP)^b^				
Likely to try it	1/4 (25)	0	1/3 (33)	1
Very likely to try it	3/4 (75)	1/1 (100)	2/3 (67)	

Injectable PrEP familiarity and acceptability among all pregnant and postpartum women	Total (*N* = 36, %)	Pregnant (*n* = 13, %)	Postpartum (*n* = 23, %)	*p*‐value
Comfort with injections				
Very comfortable	19 (53)	4 (31)	15 (65)	0.230
Comfortable	13 (36)	7 (54)	6 (26)	
Uncomfortable (I'll have an injection when I need it, but I don't like it)	3 (8)	2 (15)	1 (4)	
Very uncomfortable (I won't do it)	0 (0)	0 (0)	1 (4)	
Any experience with injectables				
Injectable contraception (e.g. Depo Provera)	17 (47)	3 (23)	14 (61)	**0.041**
Never	19 (53)	10 (77)	9 (39)	
If pregnant at time of acceptability survey: willingness to use injectable PrEP after giving birth				
Very likely		13/13 (100)	−	−

Theoretical vaginal ring acceptability among all pregnant and postpartum women^c^	Total (*N* = 36, %)	Pregnant (*n* = 13, %)	Postpartum (*n* = 23, %)	*p*‐value
Any experience with vaginal rings				
Never	35 (97)	13 (100)	22 (96)	1
Yes, previous PrEP vaginal ring	1 (3)	0 (0)	1 (4)	
Contraception	0 (0)	0 (0)	0 (0)	
Vaginal ring likes				
Nothing	29 (81)	11 (85)	18 (78)	1
Protection from HIV	3 (8)	0 (0)	3 (13)	0.633
Long‐acting	2 (6)	1 (7)	1 (4)	1
Fewer side effects	1 (3)	0 (0)	1 (4)	1
Other	3 (8)	1 (8)	1 (4)	1
Vaginal ring dislikes				
Insertion into vagina	16 (44)	7 (54)	9 (39)	0.493
Not enough safety data	7 (19)	2 (15)	5 (22)	1
My partner or I may feel it	3 (8)	1 (8)	2 (9)	1
Vaginal ring only reduces HIV risk by 30−50%	4 (11)	1 (8)	3 (13)	1
Other	6 (17)	1 (8)	5 (22)	0.385
Nothing	8 (22)	4 (31)	4 (17)	0.422
Willingness to try vaginal ring				
Very unlikely to try it	28 (78)	10 (77)	18 (78)	0.875
Unlikely to try it	3 (8)	2 (15)	1 (4)	
Still deciding	0 (0)	0 (0)	0 (0)	
Likely to try it	4 (11)	1 (8)	3 (13)	
Very likely to try it	1 (3)	0 (0)	1 (4)	
If pregnant: willingness to try vaginal ring after giving birth				
Very unlikely to try it	−	11 (85)	−	−
Unlikely to try it	−	0 (0)	−	
Still deciding	−	1 (8)	−	
Likely to try it	−	1 (8)	−	
Very likely to try it	−	0 (0)	−	
Comfort with the idea of vaginal ring insertion				
Very comfortable	0 (0)	0 (0)	0 (0)	0.398
Comfortable	3 (8)	0 (0)	3 (13)	
Uncomfortable (I'll use a vaginal ring when I need it, but I don't like it)	4 (11)	3 (23)	1 (4)	
Very uncomfortable (I won't do it)	29 (81)	10 (77)	19 (83)	

Abbreviations: CAB‐LA, long‐acting injectable cabotegravir; PrEP, pre‐exposure prophylaxis.

Bold indicates *p* < 0.05.

^a^ “Unlikely” and “Still deciding” omitted due to zero responses.

^b^ “Very unlikely,” “Unlikely” and “Still deciding” omitted due to zero responses.

^c^ Prior to asking these questions, participants were reminded that the ring is not yet approved in pregnancy or breastfeeding currently, but were asked to reflect on their perceptions and willingness to use the PrEP vaginal ring if it was approved in pregnancy and breastfeeding.

### 3.4 Theoretical acceptability of PrEP vaginal ring

Although PLP were not offered the DVR due to clinical contraindication during pregnancy and breastfeeding in South Africa, participants were provided education on the method as part of informed choice counselling at study enrolment. Of all PLP surveyed, one postpartum individual reported previous experience with a PrEP vaginal ring, while the remainder (97%, *n* = 35) described no previous experience with PrEP or contraceptive vaginal rings (Table 3). Eighty‐one percent of participants (*n* = 29) reported liking nothing about the PrEP vaginal ring, while the most frequent dislikes regarding the PrEP vaginal ring included vaginal insertion of the PrEP method (44%, *n* = 16), being unsure about its safety during pregnancy and breastfeeding (19%, *n* = 7), and its lower effectiveness in reducing HIV risk compared to oral PrEP or CAB‐LA (11%, *n* = 4). Most PLP (78%, *n* = 28) said that they were “very unlikely to try” the PrEP vaginal ring, although 14% (*n* = 5, of which four were postpartum) said they were “likely” or “very likely” to try it. Eighty‐five percent of pregnant participants surveyed (*n* = 11) said they were “very unlikely” to try the vaginal ring after giving birth.

### 3.5 Future PrEP preferences

We surveyed PLP about preferred characteristics for PrEP methods that may become available in the future (Table 4). When asked what would be the ideal duration of effectiveness of a PrEP method, 58% of PLP (*n* = 21) preferred 6 months or more, followed by 33% (*n* = 12) who wanted a method that lasts “a few months” (e.g. > 1 month). Eighty‐six percent of participants were “very comfortable” with receiving intramuscular injections. Regarding subcutaneous injections, 50% (*n* = 18) said they would not have one, although 39% (*n* = 14) still said they would either be “very comfortable with them” or “would have one in most circumstances.” The most ideal method of insertion in this sample was intramuscular injection (78%, *n* = 28), followed by subcutaneous injection (17%, *n* = 6) and two participants said that either injection would be fine. When asked who would be the ideal person to administer a PrEP injection, 86% of PLP (*n* = 31) would be comfortable with a nurse and 28% (*n* = 10) with a doctor, with one individual who would be comfortable receiving an injection from a community healthcare worker, and no preference overall for self‐administered injections. Sixty‐four percent reported never struggling to return for scheduled PrEP visits, while 17% (*n* = 6) described not getting time off from school, work or childcare, and 8% (*n* = 3) described the cost of travel to clinic (8%, *n* = 3) as barriers. The most frequently preferred locations for receiving PrEP were government clinics (67%, *n* = 24), mobile clinics (19%, *n* = 7) and home delivery (11%, *n* = 4). Fifty‐four percent of pregnant participants (*n* = 7) responded that safety during pregnancy and breastfeeding was the most important characteristic of a PrEP product, followed by its duration of protection, its effectiveness at preventing HIV and the method of delivery (e.g. injection vs. oral pill).

**Table 4:** Future pre‐exposure prophylaxis (PrEP) preferences among pregnant and postpartum women offered oral PrEP and CAB‐LA in Cape Town, South Africa, May 2024–October 2024

	Total (*N* = 36, %)	Pregnant (*n* = 13, %)	Postpartum (*n* = 23, %)	*p*‐value
Ideal PrEP duration of effectiveness				
6 months or more	21 (58)	6 (46)	15 (65)	0.676
A few months	12 (33)	6 (46)	6 (26)	
One month	3 (8)	1 (8)	2 (9)	
Daily	0 (0)	0 (0)	0 (0)	
Acceptability of intramuscular injections				
I'm very comfortable with them	31 (86)	12 (92)	19 (83)	0.988
I would have one in most circumstances	2 (6)	1 (8)	1 (4)	
I don't mind/no preference	1 (3)	0 (0)	1 (4)	
I would have one only if it was very necessary	1 (3)	0 (0)	1 (4)	
I wouldn't have one	1 (3)	0 (0)	1 (4)	
Acceptability of subcutaneous injections				
I wouldn't have one	18 (50)	8 (62)	10 (43)	0.877
I would have one only if it was very necessary	2 (6)	1 (8)	1 (4)	
I don't mind/no preference	2 (6)	0 (0)	2 (9)	
I would have one in most circumstances	3 (8)	1 (8)	2 (9)	
I'm very comfortable with them	11 (31)	3 (23)	8 (35)	
Ideal future PrEP method of insertion^a^				
Intramuscular injection	28 (78)	12 (92)	16 (70)	0.245
Subcutaneous injection	6 (17)	0 (0)	6 (26)	
Either injection would be fine	2 (6)	1 (8)	1 (4)	
Ideal person administering injection^b^				
Nurse	31 (86)	11 (85)	20 (87)	1
Doctor	10 (28)	2 (15)	8 (35)	0.270
Community healthcare worker	1 (3)	1 (8)	0 (0)	0.544
I struggle to come back to the clinic for scheduled visits because				
I never struggle to return at scheduled time	23 (64)	10 (77)	13 (57)	0.292
I can't get off school/work/childcare	6 (17)	2 (15)	4 (17)	1
Expensive to travel to clinic	3 (8)	1 (8)	2 (9)	1
I often forget appointment times	1 (3)	0 (0)	1 (4)	1
I will be travelling home or to another province to give birth	1 (3)	0 (0)	1 (4)	1
Other	4 (11)	1 (8)	3 (13)	1
Preferred location for receiving PrEP product				
Government clinic	24 (67)	11 (85)	13 (57)	0.143
Mobile truck	7 (19)	0 (0)	7 (30)	0.117
Delivered at home	4 (11)	2 (15)	2 (9)	0.609
No preference	1 (3)	0 (0)	1 (4)	1
What is the most important feature of a PrEP product for you?				
Duration of protection	13 (36)	5 (38)	8 (35)	1
Safety during pregnancy/breastfeeding	10 (28)	7 (54)	3 (13)	**0.018**
How effective it is at preventing HIV	9 (25)	1 (8)	8 (35)	0.114
Whether it has side effects/how severe they are	2 (6)	0 (0)	2 (9)	1
Ability to use product privately	1 (3)	0 (0)	1 (4)	1
Who it is given by	1 (3)	0 (0)	1 (4)	1
What is the second most important feature of a PrEP product for you?				
Duration of protection	11 (31)	4 (31)	7 (30)	1
Safety during pregnancy/while breastfeeding	8 (22)	4 (31)	4 (17)	0.422
How it is given (injection vs. oral)	6 (17)	0	6 (26)	0.219
How effective it is at preventing HIV	6 (17)	3 (23)	3 (13)	0.645
How often I need to return to the clinic/provider	1 (3)	0	1 (4)	1
Whether it has side effects/how severe they are	1 (3)	0	1 (4)	1
Ability to use product privately	2 (6)	2 (15)	0	1
Where it is given	1 (3)	0	1 (4)	1

Abbreviations: PrEP, pre‐exposure prophylaxis.

Bold indicates *p* < 0.05.

^a^ “Vaginal ring,” “Oral PrEP pill” and “Would not choose one of the above PrEP methods” omitted due to zero responses.

^b^ “Non‐medical professional,” “Self‐administered injection” and “Other” omitted due to zero responses.

## 4 DISCUSSION

Overall, PLP enrolled in an implementation study offering informed choice between oral PrEP and CAB‐LA in Cape Town, South Africa, had strong preference for CAB‐LA over oral PrEP, reported positive and negative attributes associated with CAB‐LA use, and had high willingness to continue using CAB‐LA. Our findings align with existing data on the high theoretical acceptability of CAB‐LA among PLP with experience using oral PrEP in South Africa and Kenya, and show that real‐world PrEP preferences among PLP align with previous theoretical acceptability and preference data [38]. Of note, the majority of PLP enrolled in this study had no prior PrEP use, which in conjunction with the aforementioned study suggests that preference for CAB‐LA over oral PrEP may be strong among PLP regardless of previous experience with PrEP. Despite injection‐related side effects (e.g. injection pain, injection site reactions), 96% of PLP using CAB‐LA expressed a desire to continue using the method. This aligns with existing CAB‐LA acceptability data in non‐pregnant individuals, indicating that the perceived benefits of using CAB‐LA (e.g. easier to use continuously and longer‐acting than a daily pill) may outweigh its drawbacks among PLP [39]. A modelling study showed that offering CAB‐LA to PLP in South Africa may reduce HIV incidence by 40% in this population, with downstream effects on reducing vertical transmission, which alongside our data on high CAB‐LA acceptability in this population further emphasizes the importance of increasing access to CAB‐LA among PLP.

Despite the high preference for and acceptability of CAB‐LA in our sample, some PLP still preferred to use oral PrEP when given the choice, indicating the importance of offering PLP choice in HIV prevention methods. Aligning with data from the aforementioned SEARCH trial, PLP may particularly benefit from dynamic choice models that periodically revisit the HIV prevention methods offered to the individual, as shown by the pregnant women in our study using oral PrEP who expressed interest in trying CAB‐LA, those who were interested in starting or continuing CAB‐LA after childbirth and those who expressed interest in trying the PrEP vaginal ring in the future [24]. Furthermore, as the options for HIV prevention rapidly expand, including the 6‐month lenacapavir subcutaneous injection (demonstrated 100% efficacy in preventing HIV with early pregnancy outcomes similar to the general population) as well as the 3‐month DVR (recently found to be pharmacokinetically superior to monthly DVR), it will be crucial to understand the acceptability of these methods among PLP [2, 40]. Our data show that PLP may have mixed perceptions of attributes related to upcoming PrEP methods: PLP prefer longer‐acting methods that last several months, but are less comfortable with abdominal subcutaneous injections compared to gluteal intramuscular injections, and largely do not prefer PrEP methods that need to be inserted vaginally. As these methods become available, it will be important to understand perceptions among larger numbers of PLP in diverse geographic regions and tailor rollout and educational materials accordingly. Furthermore, pregnant participants in our study most frequently reported safety during pregnancy and breastfeeding as the most important characteristic about a potential PrEP method, emphasizing the need for clear communication to potential PrEP users about safety data in pregnancy for current and upcoming PrEP methods.

Choice in HIV prevention methods alone may not be enough to optimize HIV prevention coverage among PLP: as seen with the implementation of oral PrEP in South Africa, PLP experience logistical barriers to accessing HIV prevention services, which may persist with the implementation of CAB‐LA due to the required clinical follow‐up [41, 42]. Some PLP in our study, including those using CAB‐LA, described experiencing difficulty with returning to the study site for follow‐up, and described interest in accessing HIV prevention services in mobile sites or via home delivery in addition to government clinics. As such, it will also be important to explore differentiated service delivery strategies to improve sustained access to HIV prevention services, including CAB‐LA, among PLP.

### 4.1 Limitations

Our study is limited in its sample size as well as the generalizability of its findings in settings other than Cape Town, South Africa. Given that not all PLP who had enrolled in PrEPared to Choose returned to complete the acceptability survey, we are limited in our understanding of PrEP acceptability or potential barriers to PrEP use among participants who did not attend. As presented in Figure S1, the majority of participants (*n* = 15/22) were unable to be contacted to return for an additional study visit, a limitation seen among PrEP implementation studies during pregnancy and postpartum in the region. Our findings are limited to the early acceptability of CAB‐LA but are an important contribution given the lack of data in PLP. Existing data on CAB‐LA acceptability show high acceptability among non‐pregnant users over 7 months after the first injection [39, 43]. Data collection is ongoing to evaluate whether CAB‐LA acceptability changes among PLP over an 18‐month follow‐up period (including frequency of PLP switching to other methods).

## 5 CONCLUSIONS

Overall, PLP enrolled in an ongoing implementation study offering HIV prevention services in Cape Town, South Africa, demonstrated a high real‐world preference for CAB‐LA over oral PrEP and a high willingness to continue using CAB‐LA within 3 months of initiation. HIV prevention preferences may vary among PLP, as highlighted by our findings showing both oral and injectable CAB‐LA uptake when offered PrEP choice, theoretical interest among PLP in future use of the monthly DVR and interest in future PrEP methods that are longer‐acting than CAB‐LA. This study has demonstrated that PLP would benefit from PrEP choice; increased choice may lead to enhanced PrEP uptake, as well as persistent and effective use of PrEP in this important population.

## COMPETING INTERESTS

The PrEPared to Choose study received CAB‐LA from ViiV Healthcare (London, United Kingdom). L‐GB has received honoraria for advisories to ViiV Healthcare, Gilead Sciences and Merck Pty LTD.

## AUTHORS’ CONTRIBUTIONS

The analysis presented in the manuscript was conceived by NW, DJD, CP and L‐GB. DJD, CP, ER and PM oversaw study implementation and data collection. KL and BL managed the electronic study database. NW analysed the data, wrote the first draft of manuscript and reviewed the manuscript following revision by all co‐authors. RH and CO reviewed the study design and data collection procedures and provided feedback on the manuscript draft. L‐GB, ER and CP designed the PrEPared to Choose and FastPrEP studies. All authors contributed to the development of this manuscript, and have read and approved the final manuscript.

## FUNDING

The PrEPared to Choose study was funded by the Bill and Melinda Gates Foundation and received CAB‐LA from ViiV Healthcare (London, United Kingdom).

## Supporting information




**Figure S1**: Flow diagram of pregnant and lactating people enrolled and surveyed in PrEPared to Choose, February 2024 – October 2024.

## Data Availability

The data that support the findings of this study are available upon request from the corresponding author, nwara@mednet.ucla.edu.
