# Supplementary material for: PrEP preferences and early acceptability of injectable cabotegravir among pregnant and lactating people in Cape Town, South Africa: findings from the PrEPared to Choose study
Source: J Int AIDS Soc. 2025 Jul 2;28(Suppl 2):e26492. doi: 10.1002/jia2.26492 (PMC12215810; doi:10.1002/jia2.26492)

**Supplemental Figure 1: Flow diagram of pregnant and lactating people enrolled and surveyed in PrEPared to Choose, February 2024 – October 2024.** 1800 adolescents and young people were enrolled into PrEPared to Choose. Of those, 58 were pregnant or lactating at study enrollment, and were invited to complete an acceptability survey within three months of enrollment. 36 pregnant and lactating people completed the acceptability survey, with reasons for exclusion listed in the figure.


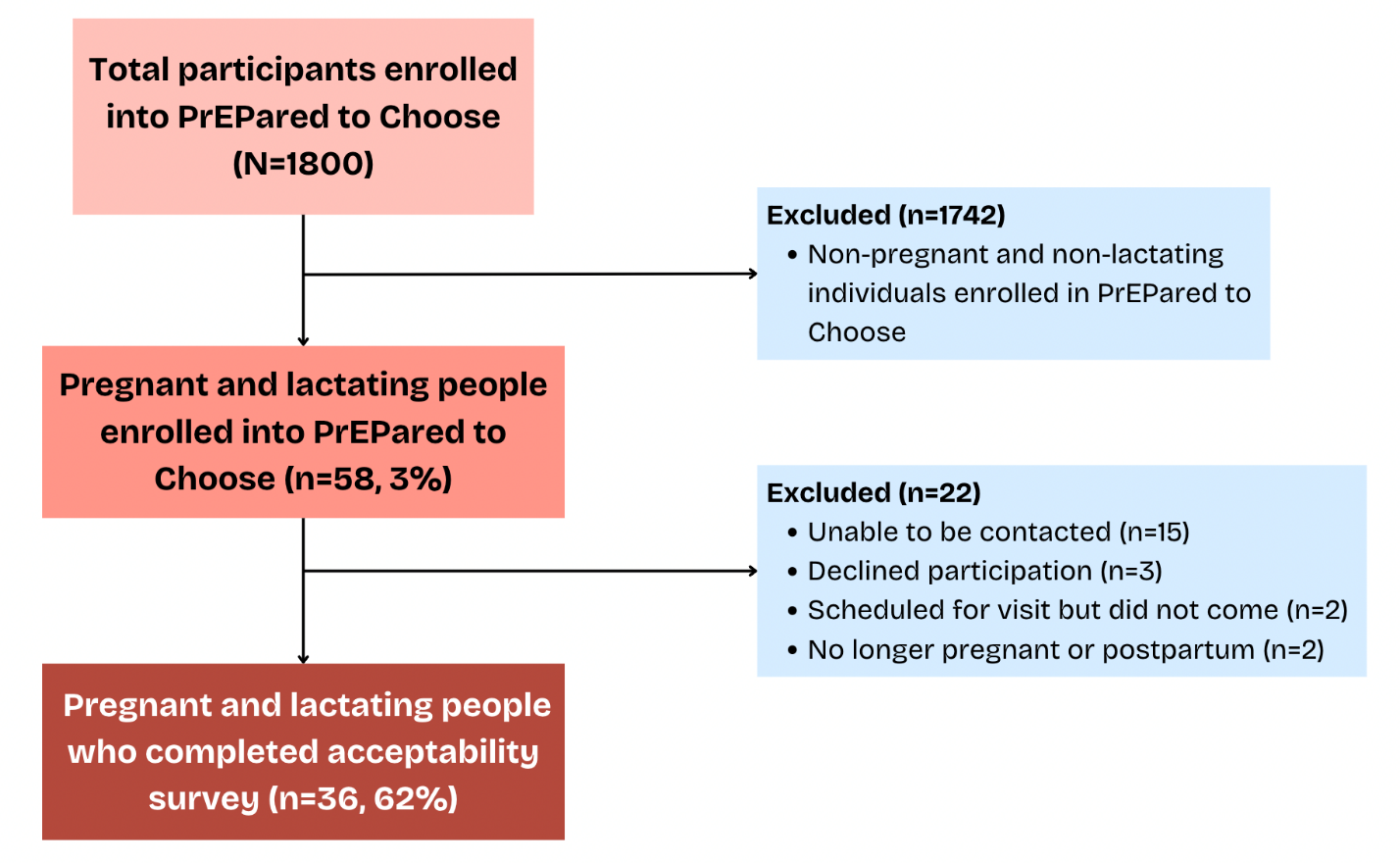

Supplement: Supplementary file 1 — Figure S1: Flow diagram of pregnant and lactating people enrolled and surveyed in PrEPared to Choose, February 2024 – October 2024. [file JIA2-28-e26492-s001.docx]
